# Toll-like receptor 4 methylation grade is linked to depressive symptom severity

**DOI:** 10.1038/s41398-021-01481-w

**Published:** 2021-06-24

**Authors:** Annica J. Rasmusson, Maike Gallwitz, Bardia Soltanabadi, Diana M. Ciuculete, Jonas Mengel-From, Kaare Christensen, Marianne Nygaard, Mette Soerensen, Adrian E. Boström, Robert Fredriksson, Eva Freyhult, Jessica Mwinyi, Darina Czamara, Elisabeth B. Binder, Helgi B. Schiöth, Janet L. Cunningham

**Affiliations:** 1 Department of Neuroscience, Psychiatry, Uppsala University, Uppsala University Hospital, Entrance 10, Floor 3B, 751 85 Uppsala, Sweden; 2 Department of Neuroscience, Functional Pharmacology, Uppsala University, BMC, Box 593, 751 24 Uppsala, Sweden; 3 The Danish Twin Registry, Epidemiology, Biostatistics and Biodemography, Department of Public Health, University of Southern Denmark, Odense, Denmark; 4 Department of Clinical Genetics, Odense University Hospital, Odense, Denmark; 5 Department of Clinical Biochemistry and Pharmacology, Odense University Hospital, Odense, Denmark; 6 Department of Pharmaceutical Biosciences, Molecular Neuropharmacology, Uppsala University, 75124 Uppsala, Sweden; 7 Department of Medical Sciences, National Bioinformatics Infrastructure Sweden, Science for Life Laboratory, Uppsala University, Uppsala, Sweden; 8 Department Translational Research in Psychiatry, Max-Planck-Institute of Psychiatry, Munich, Germany; 9 Institute of Translational Medicine and Biotechnology, I. M. Sechenov First Moscow State Medical University, Moscow, Russia

**Keywords:** Clinical genetics, Medical genetics, Human behaviour

## Abstract

This study explores potential associations between the methylation of promoter-associated CpG sites of the toll-like receptor (TLR)-family, plasma levels of pro-inflammatory proteins and depressive symptoms in young female psychiatric patients. Ratings of depressive symptoms and blood samples were obtained from 92 young women seeking psychiatric care. Methylation of 32 promoter-associated CpG sites in *TLR1* to *TLR10* was analysed using the Illumina Infinium Methylation EPIC BeadChip. Expression levels of 91 inflammatory proteins were determined by proximity extension assay. Statistical correlations between depressive state, *TLR1-10* methylation and inflammatory proteins were investigated. Four additional cohorts were studied to evaluate the generalizability of the findings. In the discovery cohort, methylation grade of cg05429895 (*TLR4*) in blood was inversely correlated with depressive symptoms score in young adults. After correction for multiple testing, plasma levels of macrophage inflammatory protein 1β (MIP-1β/CCL4) were associated with both *TLR4* methylation and depressive symptom severity. A similar inverse association between *TLR4* methylation in blood and affective symptoms score was also found in a cohort of 148 both males and females (<40 years of age) from the Danish Twin Registry. These findings were not, however, replicated in three other external cohorts; which differed from the first two cohorts by a higher age and mixed ethnicities, thus limiting the generalizability of our findings. However, *TLR4* methylation inversely correlated with *TLR4* mRNA expression in the Danish Twin Study indicating a functional significance of methylation at this particular CpG. Higher depression scores in young Scandinavian adults was associated with decreased methylation of *TLR4* in blood.

## Introduction

Major depressive disorder (MDD) is a leading contributor to the global burden of disease. It is predicted to become the foremost cause of years lost due to ill health and disability by 2030 [1]. Numerous studies point towards an induction of immune activity and pro-inflammatory pathways in unipolar and bipolar depression, mainly highlighting molecules involved in the function and regulation of the innate immune system, e.g., interleukin (IL)-1, IL-6, IL-8, C-reactive protein (CRP), tumour necrosis factor alpha (TNF-α) and interferons (IFN) [2–6]. The interaction between the brain and the immune system is both bi-directional and complex. Cytokines and chemokines associated with depressive states are suggested to directly execute both neuromodulatory and neurotransmitter-like effects and can regulate the HPA axis [7]. Immune cells produce neurotransmitters, neuroendocrine factors, such as peptide hormones, as well as their receptors, and lymphoid organs are innervated. The autonomic nerve system modulates the cytokine production via the “inflammatory reflex” and is of major importance to determine the magnitude of the inflammatory response (i.e., cytokine release from macrophages) via afferent signals from the vagal nerve to the brain. Acetylcholine interacts with nicotinic receptor α7 receptor subunit on macrophages, deactivates them and inhibits further cytokine release—thus dampening the inflammation [8].

Causal mechanisms between inflammation and depression are also suggested by induction of sickness behaviour as well as major depressive episodes in cases receiving INF-α-treatment against Hepatitis C [9,10,]. Regarding this connection between innate immunity and depression, the toll-like receptor (TLR) family is of special interest, comprising an essential part of the innate immunity [11,12,]. The ten family members are spread over endosomes and plasma and widely expressed on most white blood cells as well as many cell types in the central nervous system, acting as pattern recognition receptors (PRR) in the first line of defence against pathogens [12–14]. Previous research has implied a role in psychiatric diseases for several TLRs [15–18]. In the brain, TLRs occur among the neurons, microglia, astrocytes and oligodendrocytes [13,19,] where they are important regulators of pain and neural plasticity, development and degeneration [20]. The downstream effects of the TLRs are determined by their location and their ligand specificity, i.e., their recognition of microbe-associated molecular patterns/pathogen-associated molecular patterns (MAMPs/PAMPs) and damage-associated molecular patterns (DAMPs), which are agonists derived from pathogens or substances resulting from tissue damage [11]. Examples of such MAMPs are bacterial flagellin and lipopolysaccharide (LPS) lipoproteins, single-stranded and unmethylated DNA, and double-stranded RNA as well as elements of parasites and fungus [12]. DAMPs constitute fibrinogen, heat-shock proteins (HSP), high mobility group box protein 1 (HMGB1) and components of the extra-cellular matrix (ECM) among others [14]. Damage, stress and pathogens activate TLRs via recognition of these ligands and can initiate a variety of different processes that also have implications for the adaptive immunity.

Of particular interest in this context is TLR4, see review by Figueroa-Hall et al. [11]. This receptor is activated mainly by tissue damage and bacterial components such as lipopolysaccharide (LPS), triggering a pro-inflammatory response involving the Nuclear Factor kappa-light-chain-enhancer of activated B cells (NF-κB) pathway [21]. TLR4 acts as a hub between many neuroimmunological responses and is also thought to mediate inflammatory activity in prolonged stress and depression alongside the hypothalamic-pituitary (HPA)-axis [20,22,23,]. In line with this, both peripheral and brain TLR4 has been ascribed a pivotal role in the hypothesis that low grade, chronic neuroinflammation exacerbates depressive symptoms [24]. Elevated TLR4 mRNA and protein levels are found in brain tissue from animal models of depression [25], and in the peripheral blood of depressed patients [15,26,].

The exact mechanisms governing the expression of TLRs in psychiatric disorders are still incompletely investigated. However, one of the most studied epigenetic mechanisms, DNA methylation, has been associated to several psychiatric conditions. In patients with MDD, genes involved in stress response have been shown to be regulated by differential methylation in peripheral blood [27,28,]. A population-based study among men recently showed a positive correlation between methylation of the *TLR2* promoter in blood and hostility, and an inverse correlation with life satisfaction [29]. Differential DNA methylation in peripheral blood, rodent and post mortem human brains is also the suggested gene regulatory mechanism coupling adverse early or adult life conditions to increased risk for depression [30]. An impressive methylome-wide association (MWAS) study of both post-mortem brain tissues and different types of blood cells, comparing healthy controls with patients with MDD identified altered methylation of TLR4 in microglia and suggested that TLR activation is implicated in MDD [31]. Furthermore, elevation of cytokines in prefrontal cortex after stress are TLR4 dependent [32,33,]. In summary, TLRs are central figures in both lymphocytes and in the central nervous system for initiating the first responses to both stress and inflammation; both which are associated to depression.

Individuals with higher cytokine levels are at higher risk to develop depression [34], and blood cytokine levels differ in groupwise comparisons between psychiatric patients and healthy controls [35]. Administration of LPS to humans and rodents trigger depressive symptoms, such as anhedonia, sad mood and disturbed affective cognition [36,37,]. However, the response to both stress and LPS triggers is highly sex dependent [38] and affected by psychological traits and nocebo [39,40,]. The last decades of data have also demonstrated that differences in immune system regulation may contribute to the elevated risk of depression in females and treatment response [41,42,]. We hypothesized that differential regulation of TLRs by methylation may be altered in patients with severe depressive symptoms. Therefore, the aim of this study was to investigate whether depressive symptom severity in young adult women seeking psychiatric care is linked to methylation shifts of the TLR family. While most studies compare healthy controls with patient groups, this study is somewhat unique in its design. The immunological differences described are within a relatively homogenous population of young adult women with symptoms of depression and/or anxiety. The candidate sites were then tested in independent cohorts to evaluate the generalizability of the findings.

## Materials and methods

### Data cohort

The material and data used in this study originates from the cohort Uppsala Psychiatric Patient Samples (UPP). All data was collected from patients seeking care at the “Young Adults” section of the Department of General Psychiatry at Uppsala University Hospital in Sweden, between the years 2012 and 2014. This section provides care for patients with mainly mood and anxiety disorders, as well as neuropsychiatric and personality disorders. All new patients seeking care during that period (*n* = 623) were asked to participate in UPP and a collection of cross-sectional data and samples was gathered from those who agreed to participate (*n* = 230). The datasets from 142 female patients aged 18–25 were complete with blood samples and these were screened for this study. Patients with current pregnancy (*n* = 1), bipolar disorder (*n* = 33), history of psychotic disorders (*n* = 1), anorexia (*n* = 3), systemic inflammatory disease (*n* = 3), substance abuse (*n* = 12) and samples with inadequate DNA quality (*n* = 5) and incomplete data (*n* = 9) were excluded, i.e., in total 50 patients as some met several exclusion criteria. The remaining 92 young females were included in this study.

### Clinical data

The population characteristics for the study participants are shown in Table 1. The procedure for assessment of psychiatric diagnoses, symptoms and physical health examination upon entering UPP has been described in detail previously [43]. Diagnoses are based on the structured interviews used, the Swedish version of the M.I.N.I. International Neuropsychiatric Interview (M.I.N.I. 6.0) [44] and/or the Structural Clinical Interview for DSM IV axis I disorders (SCID-I) [45] performed by trained psychiatrists or psychologists. Severity of depressive symptoms was graded using the Montgomery Åsberg Depression Rating Scale - Self-Assessment (MADRS-S) [46], rendering 0 to 54 points. Based on MADRS-S scores, participants were arbitrarily grouped as suffering from “mild” (6–19 points), “moderate” (20–29 points) or “severe” (30+ points) depressive symptoms. Physical examination included measurement of Body Mass Index (BMI), calculated as kg/m^2^.

**Table 1:** Characteristics of the 92 female general psychiatric patients from UPP (discovery cohort) aged 18–25, grouped according to MADRS-S score.

	Mild (*n* = 32)	Moderate (*n* = 36)	Severe (*n* = 24)
MADRS-S points^a^	6–19	20–29	30+
Lifetime depressive episode	31 (96.9)	35 (95.0)	24 (100)
Current depressive episode	8 (25.0)	22 (61.1)	22 (91.7)
Any anxiety disorder	17 (53.1)	29 (80.6)	18 (75.0)
GAD	8 (25.0)	16 (44.4)	11 (45.8)
OCD	4 (12.5)	2 (5.6)	0 (0)
PTSD	1 (3.1)	6 (16.7)	4 (16.7)
Social phobia	10 (31.3)	13 (36.1)	7 (29.2)
Panic disorder	6 (18.8)	8 (22.2)	7 (29.2)
Agoraphobia	5 (15.6)	5 (13.9)	5 (20.8)
Antidepressants^b^	18 (56.3)	15 (41.7)	14 (58.3)
Smoker	8 (25.0)	11 (30.6)	9 (37.5)
Age (Mean ± SD)	20.69 ± 1.79	21.08 ± 2.09	21.25 ± 2.17
Metabolic status (Median (Q1;Q3))			
Weight	62.5 (55.8;69.1)	67.9 (54.6;79.8)	61.0 (56.9;72.6)
BMI kg/m^2^	21.4 (20.4;24.1)	24.0(20.9;27.5)	22.5 (20.0;25.3)

*MADRS-S* Montgomery Åsberg Depression Rating Scale - Self-Assessment, *GAD* generalized anxiety disorder, *OCD* obsessive-compulsive disorder, *PTSD* posttraumatic stress disorder, Q1 = 25th percentile, Q3 = 75th percentile, *SD s*tandard deviation, *UPP* Uppsala Psychiatric Patient Samples.

^a^Grouped according to severity score on MADRS-S.

^b^Use of SSRI, SNRI, Mirtazapine (Y/N).

### Initial Blood sample processing

Blood samples were obtained from non-fasting patients. Samples were collected during office hours, between the times 8:00 and 15:30, and kept in −80 °C at Uppsala Biobank. Sample collection was performed by the same person and the process for sample management was consistent for all samples. Total DNA from whole blood was extracted at the Genome Centre of the Latvian Biomedical Research and Study centre.

### Methylation profiling

Methylation profiling was performed at the SciLife Laboratory, in Uppsala, Sweden. DNA samples à 250 ng underwent bisulfite conversion using the EZ DNA Methylation—GoldTM kit (ZymoResearch, USA). According to protocol, converted DNA was eluted in 15 μl buffer and evaporated to <4 μl. The Illumina Infinium Methylation EPIC BeadChip was used for hybridisation of converted DNA, after which the array was imaged, using Illumina iScan system (Illumina, San Diego, CA, USA) – yielding a percentile quantification of methylation for each probe across the patient cohort. More than 850 K CpG sites are covered by the Infinium Methylation EPIC BeadChip. More than 90% of these sites are also represented on the Illumina Infinium 450K BeadChip, and only these CpGs were analysed.

### CpG site annotation

For CpG site annotation, we utilized the expanded annotation table by Price et al. [47]. As it was originally designed for the Illumina 450K Methylation BeadChip, only CpG sites represented also on the BeadChip were analysed. Using the annotation file, each individual CpG site was associated with a gene and the distance to the closest transcriptional start site (TSS) was calculated. Because DNA methylation and gene expression have a stronger correlation in the regions spanning 2000 base pairs (bp) up- and downstream of the TSS [48], the analysis was restricted to probes located within this region. CpG sites covering known single nucleotide polymorphism (SNP) loci or those located on sex determining chromosomes were excluded, as methylation levels of probes annotated to SNP loci are possibly affected by the genetic variants [49], and correct normalization of CpGs located on sex chromosomes have been demonstrated to be more difficult [49]. Probes that in more than >75 % displayed a detection *p* value > 10^-5^, were also excluded. We also removed cross reactive probes according to the annotation generated by Chen et al. [49].

### DNA methylation data, pre-processing and statistical corrections

Methylation data underwent pre-processing in several steps using functions taken from packages of the Bioconductor project (www.bioconductor.org, operable in R version 3.3.0) including FactoMineR, ChAMP, minfi, wateRmelon and sva. Data was subject to probe exclusion and correction for probe type as well as background correction and removal of batch effects. Pre-processing also included adjustment of the global DNA methylation pattern for white blood cell type heterogeneity [50] and a principal component analysis (PCA) to identify sample outliers.

In detail, the minfi package (v 1.18.2) for R [51] was utilized to load the original.idat files into R. Background correction was performed using the ‘preprocessNoob’ function of the minfi package in a dye-bias normalization procedure [52]. Because of the difference in dynamic range and distribution of DNA methylation pattern between the type I and type II probes on the Illumina Methylation EPIC BeadChip, adjustments were made for probe type differences using the Beta Mixture Quantile Dilation (BMIQ) function of the wateRmelon package [53]. In addition, the global DNA methylation pattern was adjusted for batch effects with the ‘ComBat’ function of the sva package [54,55,].

Because of the different cell populations in whole blood and their distinct epigenetic profiles, the global DNA methylation pattern was adjusted for white blood cell type distribution. This was achieved using a ChAMP-based statistical procedure of the Housman algorithm, implementing a reference-based approach to correct for cell-proportions in a whole blood DNA methylation dataset [50].

### Selection of CpG sites

Following the above-described pre-processing procedure, altogether 32 CpGs in promoter regions of *TLR*-family members *TLR1-10* were selected for further analysis (Supplementary table 1 for a complete list of the CpG sites in this study). Detailed information from Illumina and Price about the selected CpGs are to be found in Supplementary Table 2a–d.

### Proximity extension assay (PEA) of plasma levels of 91 inflammatory proteins

The relative levels of 91 inflammatory proteins (see Supplementary Table 3) were analysed in 92 plasma samples from the study cohort using the multiplex inflammation panel (Olink Bioscience, Sweden), a proximity extension-based assay (PEA). The 91 proteins belong to a pre-set inflammatory panel, initially including 92 proteins, but one protein brain derived neurotrophic factor (BDNF) was excluded by the manufacturer due to technical issues. In short, PEA technology allows for amplification and quantification of antibody-coupled, proximity-dependent DNA templates, reflecting relative protein levels in the sample [56,57,]. With detection sensitivity down to fg/mL, the assay can be used for comparison of relative protein values between groups but is not an absolute quantification. All samples were analysed with the same batch of reagents at the Clinical Biomarker Facility at the SciLife Lab in Uppsala. DNA amplification and quantification were carried out using the BioMark™ HD real-time PCR platform (Fluidigm, South San Francisco, CA, USA).

The 92 samples were analyzed together with 569 other plasma samples, all randomly distributed over eight plates. The samples were normalized for plate differences using a median normalization applied separately for each protein. For this normalization, we considered only the 72 proteins with detectable values in at least 80% of all the plasma samples (see Supplementary Table 3).

### Validation of plasma protein levels with electrochemiluminescence multiplex analysis

Plasma protein levels examined by the PEA technique that were significantly associated to depression grade and methylation grade after correction for multiple analysis, i.e., MIP-1β/CCL4, were validated with an electrochemiluminescence sandwich immunoassay using the Meso Scale Discovery (Rockville, MD, USA) multiplex platform.

In brief, plasma samples were applied to a primary antibody-coated 96-well plate. After subsequent dilution, incubation and washing, the captured proteins were incubated with secondary antibodies labelled with an electrochemiluminescence tag. The plate was then inserted into Sector Imager 2400 (MSD, Gaithersburg, MD). The MSD Discovery Workbench Software was used to convert the intensity of emitted light to protein concentrations (pg/mL), using interpolation from log calibrator curves, corresponding to the protein of interest. According to the manufacturer the inter-assay variation for MIP-1β/CCL4 has a mean CV of 6.1%.

### Statistical analyses

Statistical analyses were performed using the software SPSS (version 23.0) and R version 3.4.2. The degree of methylation at CpG sites was converted into β- and M-values. As M-values have been shown to be more statistically valid for analysis of differential methylation, these were used in further analyses [58]. All ordinal and linear regression analyses were adjusted for the covariates BMI, smoking (Y/N), antidepressants (Y/N), any anxiety diagnosis (Y/N), and age. We adjusted for anxiety diagnosis in the main cohort (UPP) to separate anxiety diagnoses from depressive symptoms.

### Correlation between methylation and depressive symptom grade

Methylation data for all 32 CpG sites across all 92 patient samples was used to analyse correlation between methylation and mild, moderate and severe depressive symptom grades (MADRS-S groups). For this we applied ordinal regression implemented in the polr function in the R-package MASS with depressive symptom severity as the dependent variable; and CpG methylation (M-value), anxiety, use of anti-depressive drugs, smoking, BMI and age, as independent variables. The association between CpG methylation and depressive symptom grade, adjusted for anxiety, use of anti-depressive drugs, smoking, BMI and age, was assessed using the likelihood ratio test (LRT). Results were adjusted for multiple comparisons using Benjamini-Hochberg’s false discovery rate (FDR) method with a significance threshold at FDR 5%.

### Correlation of CpG methylation with inflammatory plasma protein levels

The methylation degree of CpGs significantly correlated with depressive symptom grades was further assessed for correlation with peripheral plasma protein levels obtained by PEA analysis, using linear regression. The association between CpG methylation (M values) and protein value (NPX) was studied, for one protein and one CpG site at a time, in a linear regression model with NPX value as the dependent variable and CpG methylation and the covariates as independent variables. The association of CpG methylation with protein value after adjusting for these variables was assessed with the LRT. Results were adjusted for multiple comparisons, using Benjamini-Hochberg’s FDR method (significance threshold at FDR 5%).

### Correlation of CpG methylation and individual MADRS-S items (*Post-hoc* analysis)

The methylation degree of CpGs that correlated with MADRS-S total score was further tested for correlation with individual MADRS-S items, using ordinal regression.

### Replication of the correlation between methylation grade of *TLR4* and depressive symptom grade

We used four external cohorts to validate our findings. The one cohort most demographically similar to our subjects was the Danish twin cohort including monozygotic twins initially selected to be discordant for birth weight [59]. In the present study only young twins (30–38 years of age) were included, and 47% were females. These were sampled based on information from the Danish Twin Registry [60].

In the Danish twin study, a composite depression Affect score was computed as the sum of 9 individual items from a short version of the CAMDEX questions as previously described [61]. Higher Affect scores reflect greater levels of depression symptoms. If 2 or less items were missing, mean substitution was used for missing items, otherwise the scale was coded as missing [61]. DNA methylation levels for the cg05429895 (*TLR4*) probe were extracted from an existing methylation data set. DNA extraction, sample preparation, data processing, and DNA methylation using the Infinium Human Methylation 450K BeadChip was performed as descripted in [62]. Complete data was obtained for 140 samples. Blood leukocyte cell subtypes were counted using a Coulter LH 750 Haematology as previously described [62]. Gene expression levels for probes in the gene *TLR4* (2 probes) were extracted from an existing genome-wide transcriptome data set. Data was obtained from 142 samples. Gene expression profiling was performed using the Agilent Sure Print G3 Human GE v3 8×60K Microarray (Agilent Technologies), and RNA extraction, gene expression profiling, and data preprocessing were performed as specified in Nygaard et al. [62,63,].

Linear regression analyses were conducted with adjustment for age and gender. Post-hoc analyses additionally adjusting for cell counts showed that cell counts were not significantly associated with phenotypes and did not modify the initial associations. To adjust for the relatedness between twins, clustering was done on twin pair. Analyses were also conducted in strata of men only and women only. To compare gene expression and CpG methylation levels, pairwise correlations were estimated. All analyses were done using STATA 16.00.

The other three cohorts; The Munich Antidepressant Response Signature (MARS) cohort, the GRADY cohort and the PReDICT study, are described in detail in supplementary methods (Supplementary Materials and methods and Supplementary Table 4). Briefly, The MARS study (*n* = 206) included patients fulfilling the criteria for moderate depressive episode and were of Caucasian ethnicity. The median age was 47 years with a range of 19–79 years. The PReDICT study (*n*=321) included treatment-naïve patients who met criteria for current major depressive disorder and were of mixed ethnicity with an age range of 18–65 years. Only 38% were under the age of 34 years. The GRADY cohort (*n*=327) with mainly African American participants who were recruited as part of the GRADY Trauma Project, recruiting patients with suspected post traumatic stress disorder (Supplementary Table 4).

## Results

### *TLR4*-associated cg05429895 is less methylated in young women with high depressive symptom score

Promoter-associated CpGs of the *TLR1-TLR10* genes (*n*=32) located within 2000 bp from the closest TSS were assessed for methylation degree and compared among the three MADRS-S groups with mild, moderate and severe depressive grade. After correction for multiple testing, significant differences in methylation were identified for one *TLR4*-associated CpG (cg05429895, Table 2). Specifically, lower methylation grade of cg05429895 was found in the groups with more severe depressive symptoms (coef. = −3.05, OR=0.048, *p*=0.001, *q*=0.036, Table 2). In patients with current major depressive disorder (*n*=52) the effect size was larger (coef. = −4.23, OR 0.015, *p*=0.002). The results in Table 2 did not change when omitting the adjustment for anxiety diagnoses.

**Table 2:** Associations between methylation levels and depressive symptom groups.

Gene	CpG	Direction	*p* value	*q* value
*TLR4*	cg05429895	−3.05	0.001	0.036*
*TLR3*	cg14827929	1.28	0.068	0.79
*TLR2*	cg18652319	2.10	0.089	0.79
*TLR10*	cg24012044	1.38	0.15	0.79
*TLR5*	cg23291900	−0.81	0.21	0.79

The top five CpG sites with methylation levels most strongly association to grade of depressive symptoms are shown. Only cg05429895 (TLR4) shows a significant association with depressive symptom grade at the 5% FDR level as indicated by (*). The associations were quantified using ordinal regression adjusting for anxiety disease (Y/N), anti-depressant use (Y/N), smoking (Y/N), BMI, and age. The likelihood ratio *p* value is reported as well as the *q* value (*p* value adjusted for multiple tests).

### Methylation of *TLR4* (cg05429895) is negatively correlated with expression levels of MIP-1β/CCL4

**Fig. 1:**
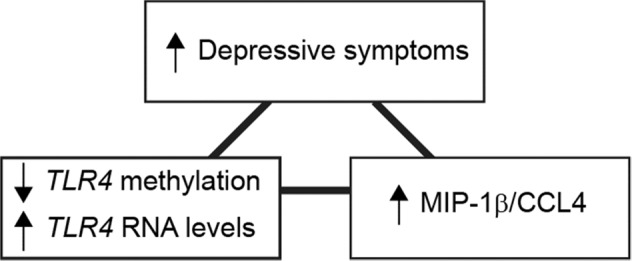
Overview of the statistical associations. Summary of the statistical associations found between depressive symptoms, methylation grade of *TLR4* cg05429895, *TLR4* RNA expression levels and MIP-1β/CCL4.

The association between relative plasma levels of 72 inflammatory proteins as analysed by PEA and the methylation status of the *TLR4*-associated cg05429895 (M-values), was calculated using an ordinal linear regression model. After adjustments for confounding factors and correction for multiple testing, a significant association was identified for only one protein: MIP-1β/CCL4 (coef.= −0.78, *p* = 0.0002, *q* = 0.015), which was negatively correlated with the methylation status of cg05429895 (*TLR4*) (Table 3, Fig. 1).

**Table 3:** Associations between methylation level of *TLR4* cg05429895 and plasma protein levels.

Protein	Coef.	*p* value	*q* value
MIP-1β/CCL4	−0.78	0.0002	0.015*
TGF-α	−0.29	0.0063	0.23
OSM	−0.71	0.022	0.52
HGF	−0.29	0.031	0.54
TNFSF14	−0.43	0.038	0.54

The top five associations between cg05429895 methylation level and plasma protein expression levels are shown. Only MIP-1β/CCL4 shows a significant association with cg05429895 (*TLR4*) methylation level at the 5% FDR level as indicated by (*).

To validate this finding, MIP-1β/CCL4 expression levels were also measured using multiplex ECL assay (Meso Scale). Using an adjusted ordered logistic regression model, MIP-1β/CCL4 levels measured by Meso Scale were shown to be associated with depression grade as well (coefficient=0.94, OR=2.55, *p* value= 0.029). The median levels were 66.1, 66.6 and 75.4 for depressive symptom groups mild, moderate, and severe, respectively.

### “Zest for life” is the one depressive item with strongest correlation to methylation grade of *TLR4* cg05429895 (*Post-hoc* analysis)

In a *post-hoc* analysis of the individual MADRS-S items, the item measuring suicidal ideation (“zest for life”) was identified as the one with the highest association with the methylation state of cg05429895 (*p*=0.002) (Supplementary Table 5).

### Replication of *TLR4*-associated cg05429895 methylation level with depressive symptom score and *TLR4* RNA expression levels

A cohort of 74 monozygotic twin pairs (*n*=148) from the Danish Twin Registry was used to validate results. The mean age was 34 years (range 30–38) and 47% were women. Affective CAMDEX scores ranged from 9 to 22 with a mean of 12. As we found in the UPP cohort, the *TLR4* methylation level was negatively associated with Affective score, and the largest effect size was here in men (coef. −65.43, *p* = 0.006) (Table 4). Interestingly, *TLR4* methylation grade correlated inversely with the RNA expression level for *TLR4* (Table 5).

**Table 4:** Associations between *TLR4* cg05429895 methylation levels and affective score, HDRS and BDI in the Danish twin study, MARS, PReDICT and GRADY cohorts.

	Danish twin study	MARS (*n* = 213)	PReDICT (*n* = 321)	GRADY (*n* = 327)
Affective score beta; *p* value				
All	−46.6; 0.005*	–	–	–
Men	−65.4; 0.0006*	–	–	–
Women	−24.2; 0.25	–	–	–
HDRS_1 beta; *p* value^#^				
All	–	0.001; 0.97	0.031; 0.13	–
Men	–	0.041; 0.35	0.078; 0.01*	–
Women	–	−0.021; 0.63	0.001; 0.96	–
HDRS_3 beta; *p* value^#^				
All	–	0.003; 0.86	−0.0156; 0.35	–
Men	–	0.024; 0.39	0.031; 0.27	–
Women	–	−0.007; 0.80	−0.040; 0.059	–
BDI_01 beta; *p* value^#^				
All	–	–	–	0.018; 0.23
Men	–	–	–	0.007; 0.82
Women	–	–	–	0.016; 0.36
BDI_09 beta; *p* value^#^				
All	–	–	–	0.006; 0.86
Men	–	–	–	0.093; 0.19
Women	–	–	–	−0.023; 0.53

Results are shown for all, and separately for men and women. # Nominal *p* value.

All associations are adjusted for age, sex (in full sample analyses) and blood cell counts. HDRS_1= Question 1 in Hamilton Depression Rating Scale (HDRS): DEPRESSED MOOD (sadness, hopeless, helpless, worthless), HDRS_3= Question 3 in HDRS: SUICIDALITY, BDI_01= Question 1 in Beck’s Depression Inventory (BDI): Sadness, BDI_09= Question 9 in BDI: SUICIDALITY.

*Significant difference, *p* < 0.05.

**Table 5:** Associations between *TLR4* cg05429895 methylation levels and *TLR4* RNA expression levels using two different RA probes in the Danish twin study.

RNA probe	Methylation level of *TLR4* cg05429895 and RNA expression
*TLR4*_A_32_P66881 beta; *p* value	−0.26; 0.002*
*TLR4*_A_24_P69538 beta; *p* value	−0.27; 0.002*

In the other three external cohorts, MARS, PReDICT and GRADY, representing a wider range of age and ethnic backgrounds compared to the initial cohort, we did not find similar associations between cg05429895 methylation level and depression scores (Table 4). These cohorts lacked data concerning somatic comorbidity.

## Discussion

This study reports an inverse correlation between methylation of *TLR4* and severity of depressive symptoms in a cohort of young adult women seeking psychiatric care mainly for symptoms of unipolar depression and/or anxiety, and this finding was replicated in a cohort of young adult twins. In both cohorts it was showed that a lower level of methylation of the *TLR4*-associated CpG site cg05429895 was associated with an increased risk of suffering from more severe depressive symptoms. We also demonstrated an association between lower methylation level of *TLR4* and higher plasma levels of MIP-1β/CCL4 in the cohort of young women. Moreover, the level of *TLR4* mRNA expression correlated to both less methylation of cg05429895 and depressive symptom severity. That both MIP-1β/CCL4 and *TLR4* RNA levels are higher in individuals with methylation of cg05429895 indicates functional significance (Fig. 1). Our findings highlight the potential role of the innate immune system, especially TLR4, in the pathophysiology of stress responses and depressive states.

A well-documented mechanism of epigenetic regulation is the silencing of gene expression by methylation of promoter-associated CpG sites [30,64,]. The importance of *TLR4* promoter methylation has previously been demonstrated in vasculitis and various malignancies, but cg05429895 has not previously been studied in the psychiatric context. However, earlier studies have associate hypomethylation of cg05429895 with increased expression of *TLR4*. For example, Yu et al. report hypomethylation of this same site to induce increased expression of TLR4 and NF-κB, leading to the development of intracranial aneurysms [65]. In gastric cancer, hypomethylation of the *TLR4* promoter also induces TLR4 expression and NF-κB signalling [66]. These findings support the hypothesis that cg05429895 hypomethylation has functional significance. We used two *TLR4* RNA probes in the Danish twin study and both probes were significantly inversely correlated to *TLR4* methylation. In a recent report by Guo et al., patients with Kawasaki disease had decreased methylation in leukocytes of cg05429895 (and two other *TLR4* CpGs) and higher mRNA *TLR4* levels [67], indicating that an increase in methylation at this particular site likely contributes to decreased RNA expression levels. Elevated *TLR4* mRNA has been described before in blood cells from depressed patients [15,26,].

We studied the association of inflammatory markers and cg05429895 hypomethylation using proximity extension assay, a new powerful technique for the simultaneous analysis of many inflammatory markers, and identified a to our knowledge novel inverse correlation between the methylation state of cg05429895 and plasma levels of MIP-1β/CCL4. Plasma levels of MIP-1β/CCL4 were verified in the same cohort using an ELISA-based method. Furthermore, MIP-1β/CCL4 levels were positively correlated with the level of depressive symptoms. Previous findings in a mouse model of depression [68] show that plasma levels of MIP-1β/CCL4 are elevated by stress and linked to elevated hippocampal mRNA expression of *TLR4* [69]. As an important chemoattractant, MIP-1β/CCL4 is secreted by several different immune cells during inflammation, including macrophages [70], and several previous reports link MIP-1β/CCL4 secretion to TLR4-stimulation [71–73]. For example, monocytes stimulated with TLR4- and TLR2 ligands increases MIP-1β/CCL4 secretion [74], and stimulation by LPS – the stereotypical TLR4 agonist – increases MIP-1β/CCL4 expression in human microglia [75]. In line with this, suppression of TLR4 in monocytes in vitro eliminates MIP-1β/CCL4 secretion [76]. Taking these earlier observations into consideration, our findings conceptualize that lower cg05429895 methylation is associated with higher *TLR4* expression and stimulation—higher MIP-1β/CCL4 expression and secretion—higher degree of depressive symptoms. A role for MIP-1β/CCL4 in depression has been reported in different contexts. Patients with chronic heart failure and MDD have elevated peripheral MIP-1β/CCL4 levels and this is more pronounced in patients with severe depression [77]. In contrast, a meta-analysis of inflammatory markers showed that MIP-1β/CCL4 are in fact lower in patients with MDD compared to healthy controls [78]. Differences in population selection, methods for the measurement of depressive symptoms or establishing the diagnosis and other factors such as age, gender and comorbidity may contribute to the discrepancies between results. We stress that the positive association between MIP-1β/CCL4 and self-rated depressive symptoms in the present study in was within the group of young adult women with diagnosis of depression and/or anxiety disorders and not a comparison with healthy controls.

There is some evidence for divergent immunological responses between men and women and the link to depressive symptoms [42,79,] as well as in their response to endotoxin-induced inflammation [42]. In the Danish twin cohort, however, the association between *TLR4* methylation and depressive symptoms was confirmed in the combined group of men and women, and there was no significant difference between men and women (data not shown). Of the four cohorts used for comparison, the Danish Twin cohort was most demographically similar to our discovery cohort with respect to age and ethnicity.

Clues for potential mechanistic explanation for the findings may be found in mouse models. Chronic stress in mice is shown to increase hippocampal TLR4 signalling even without microbial stimulus [80]. Interestingly, inhibition of TLR4 in the same model/study could prevent the development of “behavioral despair” [80]. In another study, both inhibition of TLR4 and TLR4 deficiency blocked the elevation of pro-inflammatory markers like nitric oxide synthase and cyclooxygenase 2 in prefrontal cortex in mice after stress [32,81,]. As TLR4 may be activated via PAMPs, DAMPs or XAMPs, we did an additional analysis of our data to further see if we had any indication of an ongoing LPS-driven low-grade infection. In a recent study, accepted in Psychoneuroendocrinology [82], we analyzed Lipopolysaccharide (LPS) binding protein (LBP) that may serve as a proxy for LPS [83]. LBP levels were not different between the depressive symptom groups in the initial cohort of young women (data not shown). This indicates that LPS may not be the primary suspect of the observed *TLR4* de-methylation. LPS and commensal bacteria in the large intestine down-regulates *TLR4* by hyper-methylation of the *TLR4* gene in intestinal epithelial cells [84]. Our data is more congruent with a model where TLR4 is regulated by methylation in response to chronic stress. As suggested by Chan et al, the TLR system is a strong candidate of executing the effects of acute and chronic stress via methylation in both blood and brain tissue [31]. Suicidal ideation, a marker for more severe depressive states, has in several earlier studies been linked to inflammatory processes [85–87]. Previous work has also showing significant increases of TLR4 expression in post mortem brain tissue from depressed suicide victims [88].

The complexity of the field is however illustrated by the absence of an association between lower degree of CpG methylation at cg05429895 (*TLR4*) and depressive symptom grade in three additional cohorts. Yet, we may argue that it is difficult to compare the discovery UPP cohort and these three replication cohorts, as they are not a very close match demographically, methodologically or clinically. First, the three cohorts MARS, PReDICT and GRADY not yielding replication use different scores to measure depressive symptoms. The MARS and PReDICT cohorts use the HDRS scale and the GRADY cohort use BDI scores. Even if we tried to select the two most relevant HDRS and BDI items matching those on the MADRS-S, it is still questionable if they are comparable. Second, the age range is much wider in the non-validating cohorts compared to our UPP study. Third, patients with frequent suicidal thoughts are more common in the UPP cohort indicating that it may include patients suffering from more severe depressive symptoms than the other cohorts.

Psychiatric diagnostic groups are much more symptomatically and biologically heterogeneous than many realize. Patients with the same diagnosis “depression” may have hyper or hyposomnia, hypo or hyperphagia, elevated or reduced psychomotor activity, apathy and/or agitation. The current diagnostic grouping are not sound biological constructs. As an example, we have recently demonstrated that while immunological markers are clearly elevated in young adults with primarily affective and anxiety disorders as a group compared to controls, other mechanisms may be contributing to the biological heterogeneity with in this population. A smaller subgroup of patients had high levels of autoantibodies against Lipopolysaccharide Binding Protein (LBP). These autoantibodies could inhibit LPS induction of TLR4 in vitro and the patients with these antibodies showed very low levels of both s-LBP and other pro-inflammatory markers [82].

Other limitations of this study include the cross-sectional design, which does not allow for further analysis of longitudinal variation in methylation. Samples were collected from non-fasting individuals during office hours. Circadian fluctuations in immune system regulation may have influenced the results. However, morning sampling from fasting patients does not compensate for differences in disturbed circadian rhythm in some patients and risks introducing a strong bias for including patients with higher function. As the results of this study were corrected for multiple comparisons, there is also a risk for overcorrection and loss of relevant information due to similar pathways shared by many variables in the analysis. Different proportions of white blood cells may influence global DNA methylation patterns. Data was adjusted for white blood cell type heterogeneity in the preprocessing. Linear regression with the raw data (before the adjustments for cell type heterogeneity) did not alter the result (not shown). The unclear extent to which epigenetic regulation in peripheral blood reflects epigenetic processes of the brain is another

important consideration [89,90,], and it is possible that regulation of peripheral TLR4 activity differs from that in the CNS. It is however important to recognise the findings by Chan et al. who found that the overlap between tissues (post-mortem brains and blood cells) from patients with MDD on the CpG-to-CpG level was generally low, however, they found strong evidence for the involvement of the same genes in both tissues [31]. The results imply that methylation in MDD may impact the same gene network in multiple tissues. It should also be noted that the differences in methylation between patient groups in our study are subtle (0.4–0.8%). Accumulated evidence, however, indicates that discrete methylation changes (1–5%) can confer relevant transcriptional and translational consequences especially in complex multifactorial conditions like depression or schizophrenia [91].

## Conclusions

In a population of 92 young-adult female patients, the *TLR4*-associated CpG site cg05429895 was significantly less methylated in patients with severe, as compared to mild, depressive symptoms. This finding was replicated in an external cohort of young adult twins. The lower methylation grade of cg05429895 and higher depressive symptom score were correlated with elevated *TLR4* RNA expression and elevated plasma levels of MIP-1β/CCL4, indicating a potential functional relevance. Limitations of the study may be that the generalizability of the results might be limited to young adults, or specific measures of depression symptom.

In summary, it is becoming increasingly clear that depression is a complex disease. This study relates lower levels of methylation of cg05429895, located on the first exon of *TLR4*, to depressive symptom severity and to serum levels of MIP-1β/CCL4. Importantly, this site is linked to the mRNA expression of *TLR4* in both previous work and in our replication cohort. These results are in line with the current hypothesis that innate immunity, specifically the TLR4 pathway, is connected to the aetiology and pathophysiology of depression in a subgroup of patients [92–94].

## Supplementary information

Supplementary Tables and Methods

## Data Availability

The datasets used and/or analysed during the current study are available from the corresponding author on reasonable request.
