# Supplementary material for: Toll-like receptor 4 methylation grade is linked to depressive symptom severity
Source: Transl Psychiatry. 2021 Jun 24;11:371. doi: 10.1038/s41398-021-01481-w (PMC8257733; doi:10.1038/s41398-021-01481-w)
Supplement: Supplementary file 1 — Supplementary Tables and Methods [file 41398_2021_1481_MOESM1_ESM.docx]

**SUPPLEMENTARY TABLES**

**Supplementary table 1.** List of the 32 included CpGs in the study (main cohort).

| **Gene** | **CpG** | | | | | | |
| --- | --- | --- | --- | --- | --- | --- | --- |
| *TLR1* | cg20054786 | cg22839308 |  |  |  |  |  |
| *TLR2* | cg18652319 | cg06618866 | cg06405222 | cg00000884 | cg15852258 | cg16547110 | cg19037167 |
| *TLR3* | cg12697789 | cg14827929 | cg17671280 | cg06498520 |  |  |  |
| *TLR4* | cg05429895 | cg13730105 |  |  |  |  |  |
| *TLR5* | cg21695171 | cg01181681 | cg03702975 | cg07538512 | cg20303021 | cg23291900 | cg12275981 |
| *TLR6* | cg10182418 |  |  |  |  |  |  |
| *TLR9* | cg06844837 | cg18452449 | cg21578541 | cg22484793 |  |  |  |
| *TLR10* | cg24012044 | cg17671577 | cg06935464 | cg19398783 | cg23855121 |  |  |

| **Supplementary Table 2a: Information about TLR CpGs from Illumina** | | | | | | | |
| --- | --- | --- | --- | --- | --- | --- | --- |
| CHR | MAPINFO | UCSC_REFGENE_NAME | UCSC_REFGENE_ACCESSION | ILMNID/NAME | ADDRESSA_ID | ALLELEA_PROBESEQ | INFINIUM_DESIGN_TYPE |
| 4 | 154609857 | TLR2 | NM_003264 | cg00000884 | 59727471 | TAAAACACAAATTACTCATACTATTATTATAACTTAAAAACACRTTAAAC | II |
| 1 | 223317364 | TLR5 | NM_003268 | cg01181681 | 33609351 | CAACTACTCTATAATTACACCCCAAATAACAAATAACAAAAAACTCCTCC | II |
| 1 | 223316252 | TLR5 | NM_003268 | cg03702975 | 16793436 | CAACACAAAAAAAACCCRCTCTATTTCAAAACRAAAATATAAATAACTTC | II |
| **9** | **120466640** | **TLR4** | **NR_024169;NR_024168;NM_138554** | **cg05429895** | **74741348** | **CCCTTTAACCCAAAACTACTTTAAATACACCAATTACTATAAAACRACTC** | **II** |
| 4 | 154605276 | TLR2 | NM_003264 | cg06405222 | 45601458 | CAACCRAAAAAAACTCTAAACCTCRCRCTAAATTCCAAACAAATAACCCC | II |
| 4 | 186990006 | TLR3 | NM_003265 | cg06498520 | 17642431 | ATTTTTTTTTCTATCCCTATAACCATACACTACTCAATTCTAACAACTTC | II |
| 4 | 154605087 | TLR2 | NM_003264 | cg06618866 | 15664300 | AAAAAAATACTAATTAAACACTTAACTTTCCCTATAATTACCAATCCCAC | II |
| 3 | 52262913 | TWF2 | NM_007284 | cg06844837 | 71633377 | AAAATATTTCTAAAAAATCAAAAAAACTAACAACTAAACRCACTTACAAC | II |
| 4 | 38784597 | TLR10 | NM_001017388;NM_030956 | cg06935464 | 62790333 | ACTTACACTCTCTCAACAAAACTACAAATACRTAAATAATTCRTTAACAC | II |
| 1 | 223316757 | TLR5 | NM_003268 | cg07538512 | 46650364 | AATACTAACATATATAATCTCRATAAACACTATAAAAATCTACAAAAACC | II |
| 4 | 38858536 | TLR6 | NM_006068 | cg10182418 | 21690303 | TTAAAAACAAATTAATACCATCTATTAACCCACACTAAAATTACTCCTAC | II |
| 1 | 223316272 | TLR5 | NM_003268 | cg12275981 | 73647304 | CTATTTCAAAACRAAAATATAAATAACTTCRTCTATACCRAAATCTCTTC | II |
| 4 | 186990425 | TLR3 | NM_003265 | cg12697789 | 37645305 | ATTCTACTAACAAACTCTACTCAACAAAAAAAACCCTACCCAATAAAAAC | II |
| 9 | 120466837 | TLR4 | NR_024169;NR_024168;NM_138554 | cg13730105 | 72719480 | AACCATAACCTTCCTCTCCTACATAAAACCAAAAAACTAAAAACCCTACA | I |
| 4 | 187002617 | TLR3 | NM_003265 | cg14827929 | 54613473 | TCCCTCTCATCTTTCCACCCAAATTTTCTCCTAACTTTCTTTTACTATCA | I |
| 4 | 154605258 | TLR2 | NM_003264 | cg15852258 | 66739304 | CTATCACAACCTAACTCACAATCAAAAACAAAAACCTACTAAAAAACACA | I |
| 4 | 154605262 | TLR2 | NM_003264 | cg16547110 | 15737398 | CACAACCTAACTCACAATCAAAAACAAAAACCTACTAAAAAACACAACCA | I |
| 4 | 186990202 | TLR3 | NM_003265 | cg17671280 | 19769488 | AAAAAAAAACAAAAACAATCTCTAACAACCTCAAAAAAACRTTTCAAACC | II |
| 4 | 38784881 | TLR10 | NM_030956;NM_001017388 | cg17671577 | 22779399 | ATCCCAAACATTTACRAAACTAAAAAACCCAACCTCTTACRAACTTTAAC | II |
| 3 | 52260671 | TLR9 | NM_017442 | cg18452449 | 33663500 | AATCAACTACAAACAAACCAATAAACCTAAAAAACACTCAAAAAAACAAC | II |
| 4 | 154625489 | TLR2 | NM_003264 | cg18652319 | 53650493 | ATTTTAAATATTAACAACAACAATCTCAATTTATTTTCTTTAAATTTACC | II |
| 4 | 154606177 | TLR2 | NM_003264 | cg19037167 | 43624430 | TATCCTCRCTCACTAATTACTACTAAAACRAAACTTAAATTTTAATCACC | II |
| 4 | 38784578 | TLR10 | NM_030956;NM_001017388 | cg19398783 | 74616465 | CTAAAATTCTTACCCCACRACTTACACTCTCTCAACAAAACTACAAATAC | II |
| 4 | 38799406 | TLR1 | NM_003263 | cg20054786 | 19779509 | ATATCTAATACACRCATAATCCACATACTTTACCCATCCAAAATTAACCC | II |
| 1 | 223316476 | TLR5 | NM_003268 | cg20303021 | 55714485 | CAACAACAAAAAATAAACCAAAAACCCTAAATCATAAACAACACAAAACA | I |
| 3 | 52260090 | TLR9 | NM_017442 | cg21578541 | 34766449 | CTTTTCTACCCTTATAAACACCATCTCCAAAATTCTACAAAACCTCATAC | II |
| 1 | 223307306 | TLR5 | NM_003268 | cg21695171 | 70658335 | CRAAAAACTACAAACRTCCCTAAACTAAACTCCRCTAAAAAATTCCTATC | II |
| 3 | 52261325 | TLR9 | NM_017442 | cg22484793 | 44653456 | TCCATAATACCAAACATCTAACTAACTCAACAACAAAAAACRATAACATC | II |
| 4 | 38807259 | TLR1 | NM_003263 | cg22839308 | 41779402 | CTAACTACCTCTAAAATTAATCTCAACCTACATAATATCCAATCAAAACC | II |
| 1 | 223316927 | TLR5 | NM_003268 | cg23291900 | 17759471 | CTATAATCTTCACATTTTAACCTAAAATTTAACCTACCTAAAAAAACACC | II |
| 4 | 38784938 | TLR10 | NM_030956;NM_001017388 | cg23855121 | 71797455 | CCCRCAAAAAACTAAACTTCTCAATCCCRCAAATATCTAAAACTACAAAC | II |
| 4 | 38776589 | TLR10 | NM_001017388;NM_030956 | cg24012044 | 70699368 | AAAACTACACATTATTTTACCAATAAACACAAATTTCTAAATTCTTTTAC | II |

**Supplementary Table 2b: Information about TLR CpGs from Illumina**

| FORWARD_SEQUENCE | GENOME_BUILD | SOURCESEQ | CHR_36 | COORD_36 | STRAND | PROBE_SNP | RANDOM_LOCI | METHYL27_LOCI | UCSC_REF_GROUP | PHANTOM | ENHANCER |
| --- | --- | --- | --- | --- | --- | --- | --- | --- | --- | --- | --- |
| GAACACGTCCTTAAGGCACAGATTG | 37 | CGCTTAACGTGTTCCTAA | 4 | 154829307 | R |  | NA | NA | 5'UTR |  | NA |
| CACTGTCTTGAGTTAAGACTGGTGG | 37 | AGCTGCTCTGTGATTACA | 1 | 221383987 | F |  | NA | NA | TSS1500 |  | NA |
| TGACAGCGAGACAGCACAAGAAGG | 37 | AGCACAAGAAGGACCCG | 1 | 221382875 | R |  | NA | NA | 5'UTR |  | TRUE |
| **ACTTCCTCTCACCCTTTAGCCCAGA** | **37** | **CGAGCCGCCCCACAGCA** | **9** | **119506461** | **R** |  | **NA** | **TRUE** | **Body;Body;1stExo** | **low-CpG:1195** | **TRUE** |
| CAGCCTAGCTCACGGTCAAGGGCG | 37 | CGGGGCCACCTGCCTGG | 4 | 154824726 | F | rs55742137 | NA | NA | TSS200 | high-CpG:1548 | TRUE |
| TTTGATTAGGGATTTTTTTTTCTGTC | 37 | TTTTTTTTTCTGTCCCTGT | 4 | 187227000 | R |  | NA | NA | TSS1500 |  | TRUE |
| CCCGAAGCTTCAAGAAAATACTGGT | 37 | AGAAAATACTGGTTGGG | 4 | 154824537 | R |  | NA | TRUE | TSS1500 |  | NA |
| ACGAGATGCAGGGACAGCAACAGG | 37 | CGCTGCAAGTGCGTTCAG | 3 | 52237953 | F |  | NA | NA | 3'UTR |  | NA |
| CTTACCCCACGGCTTGCACTCTCTCA | 37 | CTTGCACTCTCTCAGCAG | 4 | 38460992 | R |  | NA | NA | TSS200;TSS200 |  | NA |
| GGGTTTTCTCTGAGGGCTGTGAGA | 37 | ATGCTGGCATGTGTGATC | 1 | 221383380 | F |  | NA | NA | TSS200 |  | TRUE |
| AAAGCGGTACCTTAGAAACAGATT | 37 | CGTAGGAGTAATTCCAGT | 4 | 38534931 | R |  | NA | NA | TSS200 |  | TRUE |
| AAGGACCCGCTCTGTTTCAAAGCGA | 37 | TGTTTCAAAGCGAGGAT | 1 | 221382895 | R |  | NA | NA | 5'UTR |  | TRUE |
| TGGACTAAAGAAAAAAGGAAAGGC | 37 | TTCTGCTAGCAAACTCTA | 4 | 187227419 | F |  | NA | TRUE | 5'UTR |  | TRUE |
| GACTCTGATCCCAGCCATGGCCTTC | 37 | CGCAGGGCTCCCAGCTTT | 9 | 119506658 | R |  | NA | NA | Body;Body;1stExon | | TRUE |
| CCAGAACAGCCAAGGCTCTGGTAA | 37 | TCCCTCTCGTCTTTCCACC | 4 | 187239611 | F |  | NA | NA | Body |  | NA |
| CCCGCCTCTGGCCTGTCGCAGCCTA | 37 | CTGTCGCAGCCTAGCTCA | 4 | 154824708 | R |  | NA | NA | TSS200 | high-CpG:1548 | TRUE |
| CCTCTGGCCTGTCGCAGCCTAGCTC | 37 | CGCAGCCTAGCTCACGGT | 4 | 154824712 | R |  | NA | NA | TSS200 | high-CpG:1548 | TRUE |
| TCCAAGGATCTCAGCTTAAAAAAAG | 37 | GGAGAAAACAAAAACAA | 4 | 187227196 | F |  | NA | NA | TSS200 |  | TRUE |
| TTGTCACCCAGGCTGGAGTGCAGT | 37 | TCCCAGACATTTGCGGGA | 4 | 38461276 | F |  | NA | NA | TSS1500;TSS1500 |  | NA |
| ACTCTGGAGTCAAAGCCACAGTCCA | 37 | GTCAACTGCAAGCAGGC | 3 | 52235711 | F |  | NA | NA | TSS1500 |  | NA |
| AGACACTGGAAATTTTAGATGTTAG | 37 | CGGCAAATTCAAAGAAA | 4 | 154844939 | R |  | NA | NA | Body |  | NA |
| GGCAGGGGGTGTGTCCTCGCTCAC | 37 | CGGTGACCAAAATCCAAG | 4 | 154825627 | R |  | NA | TRUE | 5'UTR |  | TRUE |
| AACAAACAAAGCTAAGGTTCTTACC | 37 | CGCACCTGCAGCTCTGCT | 4 | 38460973 | R |  | NA | TRUE | 5'UTR;5'UTR;1stExon;1stExon | | NA |
| CCCACAATTTTCAAAAACCGTGTCT | 37 | CGGGCTAATTTTGGATGG | 4 | 38475801 | F |  | NA | NA | Body |  | NA |
| ACCCGGACAGCCCGGGAGGAGGGC | 37 | CGCCCCGCGCTGCCTACG | 1 | 221383099 | F |  | NA | NA | 5'UTR |  | TRUE |
| GCCGACTTGTCCTTTTCTGCCCTTGT | 37 | CGCATGAGGCCCTGCAG | 3 | 52235130 | R |  | NA | TRUE | 5'UTR;1stExon |  | NA |
| CGAGCGGGGCGCGGGGAGCTGCA | 37 | GGGGAGCTGCAAGCGTC | 1 | 221373929 | R |  | NA | NA | 5'UTR |  | NA |
| GGCAGGCAGGCTCCATAGTGCCAG | 37 | CCATAGTGCCAGGCATCT | 3 | 52236365 | R |  | NA | TRUE | TSS1500 |  | NA |
| TGGGATTCCAGCTGACTGCCTCTGA | 37 | TGACTGCCTCTGAAGTTG | 4 | 38483654 | R |  | NA | NA | TSS1500 |  | NA |
| TGTCCAAAAGCACAGCCAGGCCAA | 37 | CGGTGCTTCTTCAGGTAG | 1 | 221383550 | F |  | NA | NA | TSS1500 |  | NA |
| TTGCGCTAAAGCCCGCAAGAGGCT | 37 | CGCCTGTAGTCCCAGACA | 4 | 38461333 | R |  | NA | TRUE | TSS1500;TSS1500 |  | NA |
| AATTGGCTTTTGCCATCTATATTTG | 37 | CGCAAAAGAACCCAGAA | 4 | 38452984 | F |  | NA | NA | Body;Body |  | NA |

**Supplementary Table 2c: Information about TLR CpGs from Price**

| ID | MAPINFO.1 | MAPINFO.1.1 | Probe_start | Probe_end | HIL_CpG_class | HIL_CpG_Island_Name |  |  |
| --- | --- | --- | --- | --- | --- | --- | --- | --- |
| cg00000884 | 154609856 | 154609858 | 154609807 | 154609857 | LC | .;. |  |  |
| cg01181681 | 223317363 | 223317365 | 223317364 | 223317414 | LC | .;. |  |  |
| cg03702975 | 223316251 | 223316253 | 223316202 | 223316252 | HC | chr1_HCshore:223316115-223316876;chr1_ICshore:223316113-223316889 | | |
| **cg05429895** | **120466639** | **120466641** | **120466590** | **120466640** | **IC** | **.;chr9_IC:120466551-120466814** |  |  |
| cg06405222 | 154605275 | 154605277 | 154605276 | 154605326 | HC | chr4_HCshore:154604931-154605935;chr4_ICshore:154604832-154606366 | | |
| cg06498520 | 186990005 | 186990007 | 186989956 | 186990006 | LC | .;. |  |  |
| cg06618866 | 154605086 | 154605088 | 154605037 | 154605087 | HC | chr4_HCshore:154604931-154605935;chr4_ICshore:154604832-154606366 | | |
| cg06844837 | 52262912 | 52262914 | 52262913 | 52262963 | LC | .;. |  |  |
| cg06935464 | 38784596 | 38784598 | 38784547 | 38784597 | LC | .;. |  |  |
| cg07538512 | 223316756 | 223316758 | 223316757 | 223316807 | HC | chr1_HCshore:223316115-223316876;chr1_ICshore:223316113-223316889 | | |
| cg10182418 | 38858535 | 38858537 | 38858486 | 38858536 | LC | .;. |  |  |
| cg12275981 | 223316271 | 223316273 | 223316222 | 223316272 | HC | chr1_HCshore:223316115-223316876;chr1_ICshore:223316113-223316889 | | |
| cg12697789 | 186990424 | 186990426 | 186990425 | 186990475 | LC | .;. |  |  |
| cg13730105 | 120466836 | 120466838 | 120466788 | 120466838 | LC | .;. |  |  |
| cg14827929 | 187002616 | 187002618 | 187002616 | 187002666 | IC | .;chr4_IC:187002449-187002678 |  |  |
| cg15852258 | 154605257 | 154605259 | 154605209 | 154605259 | HC | chr4_HCshore:154604931-154605935;chr4_ICshore:154604832-154606366 | | |
| cg16547110 | 154605261 | 154605263 | 154605213 | 154605263 | HC | chr4_HCshore:154604931-154605935;chr4_ICshore:154604832-154606366 | | |
| cg17671280 | 186990201 | 186990203 | 186990202 | 186990252 | LC | .;. |  |  |
| cg17671577 | 38784880 | 38784882 | 38784881 | 38784931 | IC | .;chr4_IC:38784871-38785189 |  |  |
| cg18452449 | 52260670 | 52260672 | 52260671 | 52260721 | LC | .;. |  |  |
| cg18652319 | 154625488 | 154625490 | 154625439 | 154625489 | LC | .;. |  |  |
| cg19037167 | 154606176 | 154606178 | 154606127 | 154606177 | ICshore | .;chr4_ICshore:154604832-154606366 |  |  |
| cg19398783 | 38784577 | 38784579 | 38784528 | 38784578 | LC | .;. |  |  |
| cg20054786 | 38799405 | 38799407 | 38799406 | 38799456 | LC | .;. |  |  |
| cg20303021 | 223316475 | 223316477 | 223316475 | 223316525 | HC | chr1_HCshore:223316115-223316876;chr1_ICshore:223316113-223316889 | | |
| cg21578541 | 52260089 | 52260091 | 52260040 | 52260090 | LC | .;. |  |  |
| cg21695171 | 223307305 | 223307307 | 223307256 | 223307306 | IC | .;chr1_IC:223306625-223307670 |  |  |
| cg22484793 | 52261324 | 52261326 | 52261275 | 52261325 | LC | .;. |  |  |
| cg22839308 | 38807258 | 38807260 | 38807209 | 38807259 | LC | .;. |  |  |
| cg23291900 | 223316926 | 223316928 | 223316927 | 223316977 | LC | .;. |  |  |
| cg23855121 | 38784937 | 38784939 | 38784888 | 38784938 | IC | .;chr4_IC:38784871-38785189 |  |  |
| cg24012044 | 38776588 | 38776590 | 38776589 | 38776639 | LC | .;. |  |  |

**Supplementary Table 2d: Information about TLR CpGs from Price**

| Closest_TSS | Closest_TSS_1 | Distance_closest_TSS | Closest_TSS_gene_name | Closest_TSS_Transcr | SPOT_ID |
| --- | --- | --- | --- | --- | --- |
| 154609053 | 154609054 | 804 | TLR2 | AF051152 | cg00000884 |
| 223316623 | 223316624 | -740 | TLR5 | NM_003268 | cg01181681 |
| 223316623 | 223316624 | 372 | TLR5 | NM_003268 | cg03702975 |
| **120466459** | **120466460** | **181** | **TLR4** | **NR_024169** | **cg05429895** |
| 154605440 | 154605441 | -164 | TLR2 | BC033756 | cg06405222 |
| 186990308 | 186990309 | -302 | TLR3 | NM_003265 | cg06498520 |
| 154605440 | 154605441 | -353 | TLR2 | BC033756 | cg06618866 |
| 52263797 | 52263798 | 885 | TLR9 | AF246974 | cg06844837 |
| 38784610 | 38784611 | 14 | TLR10 | NM_001017388 | cg06935464 |
| 223316623 | 223316624 | -133 | TLR5 | NM_003268 | cg07538512 |
| 38858437 | 38858438 | -98 | TLR6 | NM_006068 | cg10182418 |
| 223316623 | 223316624 | 352 | TLR5 | NM_003268 | cg12275981 |
| 186990308 | 186990309 | 117 | TLR3 | NM_003265 | cg12697789 |
| 120466459 | 120466460 | 378 | TLR4 | NR_024169 | cg13730105 |
| 187002770 | 187002771 | -153 | TLR3 | AK302143 | cg14827929 |
| 154605440 | 154605441 | -182 | TLR2 | BC033756 | cg15852258 |
| 154605440 | 154605441 | -178 | TLR2 | BC033756 | cg16547110 |
| 186990308 | 186990309 | -106 | TLR3 | NM_003265 | cg17671280 |
| 38784610 | 38784611 | -270 | TLR10 | NM_030956 | cg17671577 |
| 52260178 | 52260179 | -492 | TLR9 | NM_017442 | cg18452449 |
| 154624059 | 154624060 | 1430 | TLR2 | CCDS3784 | cg18652319 |
| 154605440 | 154605441 | 737 | TLR2 | NM_003264 | cg19037167 |
| 38784610 | 38784611 | 33 | TLR10 | NM_001017388 | cg19398783 |
| 38800451 | 38800452 | 1046 | TLR1 | CCDS33973 | cg20054786 |
| 223316623 | 223316624 | 148 | TLR5 | NM_003268 | cg20303021 |
| 52260178 | 52260179 | 89 | TLR9 | NM_017442 | cg21578541 |
| 223308205 | 223308206 | 900 | TLR5 | BC109119 | cg21695171 |
| 52260178 | 52260179 | -1146 | TLR9 | NM_017442 | cg22484793 |
| 38806411 | 38806412 | -847 | TLR1 | NM_003263 | cg22839308 |
| 223316623 | 223316624 | -303 | TLR5 | NM_003268 | cg23291900 |
| 38784610 | 38784611 | -327 | TLR10 | NM_030956 | cg23855121 |
| 38777210 | 38777211 | 622 | TLR10 | CCDS3445 | cg24012044 |

**Supplementary table 3.** List of proteins included in the Proximity extension assay PEA Multiplex inflammation panel. The statistical analysis included 72 of 91 proteins where detectable values were present in at least 80% of all the plasma samples. Brain derived neurotrophic factor (BDNF) was initially included in the assay, but manufacturer (O-Link) excluded it because of technical issues. The excluded 19 proteins are indicated with strike through.

Adenosine Deaminase (ADA)

~~Artemin (ARTN)~~

Axin-1 (AXIN1)

C-C motif chemokine 19 (CCL19)

C-C motif chemokine 20 (CCL20)

C-C motif chemokine 23 (CCL23)

C-C motif chemokine 25 (CCL25)

C-C motif chemokine 28 (CCL28)

C-C motif chemokine 3 (CCL3)

C-C motif chemokine 4 (CCL4 )

C-X-C motif chemokine 1 (CXCL1)

C-X-C motif chemokine 10 (CXCL10 )

C-X-C motif chemokine 11 (CXCL11)

C-X-C motif chemokine 5 (CXCL5 )

C-X-C motif chemokine 6 (CXCL6)

C-X-C motif chemokine 9 (CXCL9 )

Caspase-8 (CASP-8 )

CD40L receptor (CD40)

CUB domain-containing protein 1 (CDCP1)

Cystatin D (CST5)

Delta and Notch-like epidermal growth factor-

related receptor (DNER)

Eotaxin (CCL11)

Eukaryotic translation initiation factor 4E-

binding protein 1 (4E-BP1)

Fibroblast growth factor 19 (FGF-19)

Fibroblast growth factor 21 (FGF-21)

Fibroblast growth factor 23 (FGF-23)

Fibroblast growth factor 5 (FGF-5)

Fms-related tyrosine kinase 3 ligand (Flt3L)

Fractalkine (CX3CL1 )

Glial cell line-derived neurotrophic factor (GDNF)

Hepatocyte growth factor (HGF)

~~Interferon gamma (IFN-gamma)~~

~~Interleukin-1 alpha (IL-1 alpha)~~

Interleukin-10 (IL10)

~~Interleukin-10 receptor subunit alpha (IL-10RA)~~

Interleukin-10 receptor subunit beta (IL-10RB)

Interleukin-12 subunit beta (IL-12B)

~~Interleukin-13 (IL-13)~~

Interleukin-15 receptor subunit alpha (IL-15RA)

Interleukin-17A (IL-17A)

Interleukin-17C (IL-17C)

Interleukin-18 (IL-18)

Interleukin-18 receptor 1 (IL-18R1)

~~Interleukin-2 (IL-2)~~

~~Interleukin-2 receptor subunit beta (IL-2RB)~~

~~Interleukin-20 (IL-20)~~

~~Interleukin-20 receptor subunit alpha (IL-20RA)~~

~~Interleukin-22 receptor subunit alpha-1 (IL-22 RA1)~~

~~Interleukin-24 (IL-24)~~

~~Interleukin-33 (IL-33)~~

~~Interleukin-4 (IL-4)~~

~~Interleukin-5 (IL5)~~

Interleukin-6 (IL6)

Interleukin-7 (IL-7)

Interleukin-8 (IL-8)

Latency-associated peptide transforming

growth factor beta-1 (LAP TGF-beta-1)

~~Leukemia inhibitory factor (LIF)~~

Leukemia inhibitory factor receptor (LIF-R)

Macrophage colony-stimulating factor 1 (CSF-1)

Matrix metalloproteinase-1 (MMP-1)

Matrix metalloproteinase-10 (MMP-10)

Monocyte chemotactic protein 1 (MCP-1)

Monocyte chemotactic protein 2 (MCP-2)

~~Monocyte chemotactic protein 3 (MCP-3)~~

Monocyte chemotactic protein 4 (MCP-4)

Natural killer cell receptor 2B4 (CD244)

Neurotrophin-3 (NT-3)

~~Neurturin (NRTN)~~

Oncostatin-M (OSM)

Osteoprotegerin (OPG)

Programmed cell death 1 ligand 1 (PD-L1)

Protein S100-A12 (EN-RAGE )

Signaling lymphocytic activation molecule (SLAMF1)

SIR2-like protein 2 (SIRT2)

STAM-binding protein (STAMPB)

Stem cell factor (SCF)

Sulfotransferase 1A1 (ST1A1)

T cell surface glycoprotein CD6 isoform (CD6)

T-cell surface glycoprotein CD5 (CD5)

~~Thymic stromal lymphopoietin (TSLP)~~

TNF-beta (TNFB)

TNF-related activation-induced cytokine (TRANCE)

TNF-related apoptosis-inducing ligand (TRAIL)

Transforming growth factor alpha (TGF-alpha)

Tumor necrosis factor (Ligand) superfamily,

member 12 (TWEAK)

~~Tumor necrosis factor (TNF)~~

Tumor necrosis factor ligand superfamily

member 14 (TNFSF14 )

Tumor necrosis factor receptor superfamily

member 9 (TNFRSF9)

Urokinase-type plasminogen activator (uPA)

Vascular endothelial growth factor A (VEGF-A)

**Supplementary table 4.** Characteristics of MARS, PReDICT and GRADY cohorts. ^§^ Only those individuals with methylation data are included in this study

|  | **MARS** | **PReDICT** | **GRADY** |
| --- | --- | --- | --- |
| N (females/males) **^§^** | 206 (109/97) | 321 (185/136) | 327 (243/84) |
| Age | median 47 (19-79) | (18-65), 38% 18-34 years | mean (SD) 32 (8) |
| Ethnicity (Country) | European (Munich) | Multi-ethnical (USA) | African American (USA) |
| Depressive instrument | Hamilton Depression Rating Scale (HDRS) | Hamilton Depression Rating Scale (HDRS) | Beck's Depression Inventory (BDI) |

**Supplementary Table 5**. Associations between methylation level of cg05429895 (*TLR4*) and the individual MADRS-S items. (*) denotes associations that are considered significant, with a FDR less than 5%. ^a^”Appetite” measure loss of appetite and not hyperphagia. ^b^”Sleep” measures insomnia and not hypersomnia.

| MADRS-S item | Direction | *p*-value |
| --- | --- | --- |
| Zest for life | -2.63 | **0.002*** |
| Mood | -2.44 | **0.006*** |
| Emotional involvement | -2.22 | **0.007*** |
| Pessimistic thoughts | -1.94 | **0.024*** |
| Initiative | -1.86 | **0.025*** |
| Ability to concentrate | -1.78 | **0.050*** |
| Appetite^a^ | -1.21 | 0.130 |
| Sleep^b^ | -1.08 | 0.160 |
| Feelings of unease | -0.95 | 0.250 |

**Supplementary Materials and methods:**

The Munich Antidepressant Response Signature (MARS) cohort included patients within 1–3 days of admission to the hospital and diagnosis was ascertained according to the Diagnostic and Statistical Manual of Mental Disorders (DSM) IV criteria. Briefly, patients fulfilling the criteria for at least a moderate depressive episode (HAM-D ≥ 14 on the 21-item Hamilton Depression Rating Scale) entered the analysis. Patients suffered from a first depressive episode or from a recurrent depressive disorder. Patients were severely depressed at admission, scoring 27.0 points in the HAM-D_21_ (median) and had already suffered from depression on average for about 40 weeks prior to admission. The samples for selected for methylation analysis were taken at admission. All included patients were of European descent. The study is described in detail in (92).

The PReDICT consists of treatment-naïve patients who met criteria for current major depressive disorder (MDD) as defined in the Diagnostic and Statistical Manual of Mental Disorders, 4th Edition (DSM-IV). HDRS-17 scores ≥18 at screening and ≥15 at the baseline visit are to be randomly assigned equally to one of three possible treatments: (1) a selective serotonin reuptake inhibitor (SSRI, escitalopram); (2) a serotonin norepinephrine reuptake inhibitor (SNRI, duloxetine); or (3) individual cognitive behavior therapy (CBT). The samples for selected for methylation analysis were taken at admission/baseline. All participants were recruited in three Atlanta sites associated with the Emory University School of Medicine, Department of Psychiatry and Behavioural Sciences (93).

The GRADY cohort consists of mainly African American (94%) participants who were recruited as part of the GRADY Trauma Project at the Grady Memorial Hospital in Atlanta, Georgia (94). All participants come from an urban population with low socioeconomic status and are characterized by high prevalence and severity of trauma over lifetime where 87.8% had experienced any significant trauma as serious accident (47%) or natural disaster (22%). Of the study population the individuals had the following: Psychiatric hospitalization (15%), involuntary psychiatric hospitalization (5%) and Suicide Attempt (13%). A majority of the participants suffered from Post-traumatic stress syndrome (PTSD). All studies were approved by the local ethics committes.

In all three cohorts, DNA methylation was measured by Illumina Infinium HumanMethylation450K BeadChips. After quality control, beta values were normalized using functional normalization (54). Batch effects were removed using ComBat with the sva package. Subsequently, all CpGs on sex chromosomes and CpGs with SNPs in the probe sequence were removed. Additionally, probes were removed if the detection *p*-value was > 0.01 in at least 25% of the samples, the probe contained SNPs in the single base pair (bp) extension or CpG position, the probe had missing beta values, or was a cross-reactive probe (49). Probe positions were reannotated and cross-hybridizing probes were removed. The Houseman method was used to estimate cell type composition (50).

All statistical analyses were conducted in R 3.5.2. We assessed association between m-values of *TLR4* cg0542989 and depression severity (HDRS items 1 and 3 in MARS and PReDICT, BDI items 1 and 9 in GRADY) using linear regression models and adjusting for age, gender and blood cell counts. In PReDICT, we additionally included the first two principal components as it is a multi-ethnical cohort.
